# Effectiveness of Platelet-Rich Plasma Therapy in Androgenic Alopecia—A Meta-Analysis

**DOI:** 10.3390/jpm12030342

**Published:** 2022-02-24

**Authors:** Simona Roxana Georgescu, Andreea Amuzescu, Cristina Iulia Mitran, Madalina Irina Mitran, Clara Matei, Carolina Constantin, Mircea Tampa, Monica Neagu

**Affiliations:** 1 Department of Dermatology, “Carol Davila” University of Medicine and Pharmacy, 020021 Bucharest, Romania; simonaroxanageorgescu@yahoo.com (S.R.G.); matei_clara@yahoo.com (C.M.); 2 Department of Dermatology, “Victor Babes” Clinical Hospital for Infectious Diseases, 030303 Bucharest, Romania; 3 Department of Microbiology, “Carol Davila” University of Medicine and Pharmacy, 020021 Bucharest, Romania; cristina.iulia.mitran@gmail.com (C.I.M.); madalina.irina.mitran@gmail.com (M.I.M.); 4 Immunology Department, “Victor Babes” National Institute of Pathology, 050096 Bucharest, Romania; caroconstantin@gmail.com (C.C.); neagu.monica@gmail.com (M.N.); 5 Colentina Clinical Hospital, 020125 Bucharest, Romania; 6 Faculty of Biology, University of Bucharest, 76201 Bucharest, Romania

**Keywords:** androgenic alopecia, clinical trial, hair density, inflammation, meta-analysis, platelet-rich plasma

## Abstract

Platelet-rich plasma (PRP) represents a novel therapy tested and is used more and more frequently in dermatology and cosmetic surgery for a variety of conditions, including androgenic alopecia (AGA), a common condition with a complex pathogenesis involving genetic factors, hormonal status and inflammation. We performed an extensive literature search which retrieved 15 clinical trials concerning the use in AGA of PRP therapy, alone or in combination, in male, female or mixed patient groups. A quantitative statistical meta-analysis of *n* = 17 trial groups proved significant increases in hair density from 141.9 ± 108.2 to 177.5 ± 129.7 hairs/cm^2^ (mean ± SD) following PRP (*p* = 0.0004). To the best of our knowledge, this is the first meta-analysis that proved a statistically significant correlation between the number of PRP treatments per month and the percentage change in hair density (*r* = 0.5, *p* = 0.03), as well as a negative correlation between the mean age of treatment group and the percentage change in hair density (*r* = −0.56, *p* = 0.016). Other factors considered for analysis were the PRP preparation method, amount used per treatment, hair diameter, terminal hairs and pull test. We conclude that PRP represents a valuable and effective therapy for AGA in both males and females if patients are rigorously selected.

## 1. Introduction

Alopecia is a common condition that affects a large part of the population, particularly Caucasian males, the most common type being androgenic alopecia (AGA), a progressive disorder with significant psychosocial effects that can lead to depression. There are several theories trying to explain the complex multifactorial pathogeny of this condition. One of them postulates that chronic perifollicular microinflammation amplifies the expression of pro-inflammatory cytokines, leading to oxidative stress. The oxidative profile acts in combination with high levels of androgens, genetic predisposition and environmental factors such as stress, affecting the corticotropin-releasing hormone pathway and cortisol levels, hence generating the condition [1]. Gene variants that lead to increased activity of 5-α-reductase or increased sensitivity of androgen receptors are deemed to play an important role [2]. There is no single key mechanism involved in the disease, but a plethora of interconnected mechanisms that need to be treated simultaneously for a successful result. The reestablishment of healthy hair cell follicles can be influenced by certain growth factors that can stimulate the anagen phase of hair cycle, favoring cell proliferation, improvement of extracellular matrix and neoangiogenesis [3,4]. Recent studies have evidenced inflammatory infiltrates located in perifollicular/perivascular areas with histiocytes and lymphocytes. These findings, together with the implication of certain inflammatory genes (e.g., CASP7, TNF), strongly suggest a relationship between AGA and inflammatory pathways [1].

The close relationship between chronic inflammation and oxidative stress is well known [5,6,7,8]. There are many studies that show the involvement of oxidative stress in alopecia areata [9,10,11,12]. Recent research indicates an imbalance between oxidants and antioxidants in AGA [13,14]. The skin appendages, especially the hair follicle, can be exposed to elevated levels of oxidative stress. Reactive oxygen species (ROS) accumulate in the hair follicle and the antioxidant defense capacity is exceeded, resulting in premature aging of dermal papilla cells and eventually to the loss of their function [13,15]. Furthermore, it has been hypothesized that ROS may mediate the response of cells to growth factors [13]. In the light of oxidative stress involvement in AGA, Kaya Erdogan et al. consider that antioxidant therapies may be useful in the treatment of these patients [14]. 

Beyond classical AGA therapies such as minoxidil or finasteride, several new strategies have been proposed in recent years, each of them claiming long-term beneficial effects on hair follicle homeostasis [1,16], and offering multiple choices for personalized treatments. Among these new therapies, platelet-rich plasma (PRP) has been tested in several randomized case-control clinical trials [17,18]. The procedure involves collecting autologous blood from the patient under sterile conditions, on anticoagulant, and separating a platelet-rich plasma fraction by centrifugation. After a first spin, the second intermediate layer of plasma can be collected with a sterile syringe, dropping out the first layer of PPP (Platelet-Poor Plasma or buffy coat) and the red blood cells at the bottom of the tube. Some practitioners use a second spin to concentrate the PRP, as well as an activator of platelet α-granules release such as calcium or thrombin before injection into the scalp. There is a wide variation of protocols for preparation of PRP [3].

PRP therapy is already extensively used in dermatology and plastic surgery [19], being successfully applied for facial rejuvenation, treatment of wrinkles, scars, striae distensae, atrophic acne, vitiligo, facelift surgery, periorbital rejuvenation, dermal augmentation, as well as in other medical specialties such as orthopedic and trauma surgery, ocular surgery, stomatology, wound healing, urology, etc. [20,21]. Due to the important effects of growth factors present in PRP on skin rejuvenation, fibroblast and macrophage chemotaxis, fibroblast proliferation and extracellular matrix synthesis [22], this treatment is expected to exert beneficial effects on hair follicle degeneration, particularly in AGA. Autologous platelet rich fibrin (PRF), another related therapy, has been successfully used in both solid and liquid form administered intralesionally to accelerate healing in a case of facial pyoderma gangrenosum, due to its capacity to promote the prolonged release of multiple growth factors including TGF, EGF, FGF, KGF, CTGF, TNF-α [23], as well as in oral and maxillofacial surgery to cure medication-related osteonecrosis [24,25].

Until now, the only two effective treatments of AGA approved by the FDA are minoxidil for both men and women and finasteride for men [26,27,28]. Other treatment options are low-level laser therapy or hair transplant surgery, both with limited effectiveness, as well as synthetic PRP analogues such as growth factors biomimetic cocktails [16] or hair follicle dermal papilla stem cell cloning [29]. Considering their potential adverse reactions and other limiting factors, published results of clinical trials have to be carefully assessed via meta-analyses like the present one, which can be expanded for finding new possible therapy targets.

## 2. Materials and Methods

### 2.1. Literature Search and Selection of Studies

A search of the literature was performed by interrogating the PubMed database (on 27 July 2021) with the keyword combination “PRP AND alopecia AND clinical trial”, a sufficiently specific search string that avoids sampling biases. We selected for meta-analysis the clinical trials that included treatment groups, using as therapy PRP alone, combined with or compared to other procedures. The exclusion criteria comprised clinical trials including patients with other types of alopecia (e.g., alopecia areata, seborrheic dermatitis, iatrogenic alopecia, *a*.*s*.*o*.). The stepwise procedure for selecting clinical trials included in the meta-analysis observed the methods, stages, as well as selected checklist items of the PRISMA (Preferred Reporting Items for Systematic Reviews and Meta-Analyses) guidelines and its subsequent updates [30,31]. 

### 2.2. Data Analysis

A number of qualitative or quantitative assessment variables (e.g., hair density, diameter, results of pull test, total number and frequency of PRP treatments, total amount of PRP per treatment, mean age of each group) were retrieved from each published clinical trial included in this meta-analysis. Data are expressed as means ± SD. The normality of the data samples was verified with the Kolmogorov-Smirnov, D’Agostino-Person and Shapiro-Wilk tests; normally distributed data were compared with Student’s *t* test, and data sets that did not pass normality tests with its non-parametric versions: Mann-Whitney for independent data sets and Wilcoxon signed rank test for paired data. Most variables included in analysis were normally distributed, therefore we used directly Student’s *t* test for independent samples (two-tailed), except for the absolute change in hair density, where we used its non-parametric variant, the non-directional Mann-Whitney test. We also performed linear correlation and regression analysis, indicating the values of Pearson’s product-momentum correlation coefficient *r*, its statistical significance *p*, and the linear regression function. Statistical significance was assessed using a critical level *p* = 0.05. Forrest plots were generated with the clinical trials meta-analysis software Review Manager (RevMan) Version 5.4.1 The Cochrane Collaboration, 2020 [32].

## 3. Results

**Figure 1:**
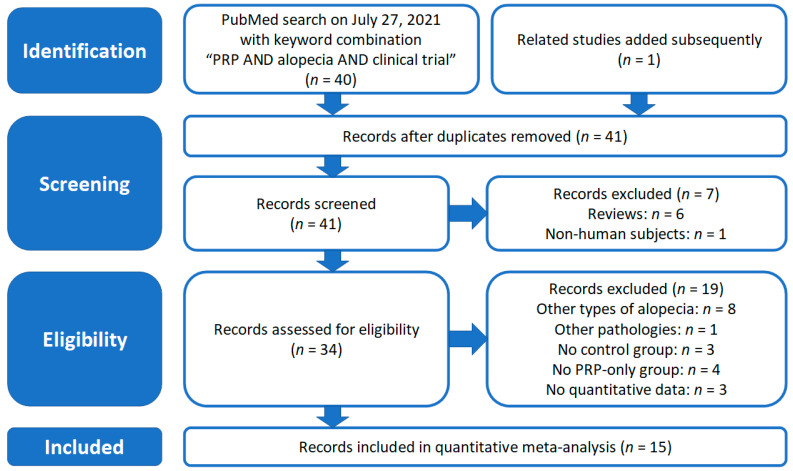
PRISMA flowchart showing the algorithm used for selection of the studies included in the current meta-analysis.

The literature search performed according to the criteria described within the Methods section resulted in the identification of 15 clinical trials, including studies on the effects of PRP treatment of AGA suitable for inclusion in our meta-analysis. The PRISMA flowchart describing the successive steps of the selection procedure is shown in Figure 1, while Table 1 presents the main features of the selected clinical trials. Most of them (86.7%) were randomized, 46.7% were double-blind, and 26.7% were single-blind. We found marked differences regarding the study design: 2 studies applied PRP/placebo on half-scalp and 3 studies on individual areas of the scalp in same patients, resulting in better case-control matching. Two studies included separate PRP and negative control (placebo) groups, while two other studies comprised two groups with different PRP administration protocols, differing in timing or preparation quality; three studies compared effects of PRP alone with those of PRP combined with minoxidil, or different PRP/minoxidil combinations, or other combinations of synthetic growth factors similar to those retrieved in PRP [16].

**Table 1:** Features of the clinical trials selected and included in the meta-analysis (in alphabetical order).

Study	Rando- Mized	Double- Blind	Groups	Mean Age &/Age Interval	Sex	mL PRP/ Treatment	Change in Hair Density (Hairs/cm^2^) Initial–Final
Alves & Grimalt 2016 [26]	Y	Y	*n* = 22, PRP/placebo on half-scalp 3 PRP treatments (1/month)	39 (21–62)	M/F	3	165.7–179.9
Bayat et al. 2019 [33]	N	N	*n* = 19, 3 PRP treatments (0, 4, 8 weeks)	36.26 (28–40)	M	5	30.11–38.58
Bruce et al. 2020 [34]	Y	N	arm A–PRP then minoxidil (*n* = 19) 3 PRP treatments over 4 weeks 8 weeks wash-out then minoxidil 12 weeks	56 (20–79)	F	5	134–145
			arm B–minoxidil then PRP (*n* = 18) minoxidil 12 weeks, 8 weeks wash-out then 3 PRP treatments over 4 weeks	56 (20–79)	F	5	139–153
Butt et al. 2018 [35]	N	N	*n* = 30, 2 PRP treatments (1/month)	28.7 (19–47)	M/F	4	34.18–50.2
Butt et al. 2019 [36]	Y	N	PRP group (*n* = 11) 2 PRP treatments at 4 weeks	26.45	M/F	5	52.64–63.72
			stromal vascular fraction-PRP group (*n* = 11), same treatment timing	33.27	M/F	5	37.66–57.11
Gentile et al. 2015 [37]	Y	Y	*n* = 23, PRP/placebo on half-scalp 3 PRP treatments (1/month)	34.74 (19–63)	M	9	161.2–207.1
Hausauer & Jones 2018 [38]	Y	single-blind	PRP group 1 (*n* = 20) 3 PRP treatments (1/month) + 1 booster treatment after 3 months	40.1 (18–60)	M/F	5	160.4–207.1
			PRP group 2 (*n* = 19) 2 PRP treatments at 3 months	46.85 (18–60)	M/F	5	177.6–190.6
Kapoor et al. 2020 [16]	Y	single-blind	group B (PRP) (*n* = 25) 8 PRP treatments at 3 weeks	25–50	M	1.5	167.2–176.1
Mapar et al. 2016 [39]	Y	single-blind	*n* = 17, PRP/placebo on squares 2 PRP treatments (1/month)	37.2 (25–45)	M	1.5	87.29–85.06
Pakhomova & Smirnova 2020 [40]	Y	single-blind	group I (PRP) (*n* = 25) 4 PRP treatments (1/month)	29.7 (18–53)	M	4	381.5–426.1
			group II (PRP + minoxidil) (*n* = 22) 4 PRP treatments (1/month)		M	4	408.4–539.6
Puig et al. 2016 [41]	Y	Y	PRP group (*n* = 15) 1 treatment	>18	F	10	no signifcant differences
			placebo group (*n* = 11) 1 treatment				
Rodrigues et al. 2020 [42]	Y	Y	PRP group (*n* = 15) 4 treatments at 15 days intervals	32 (18–50)	M	2	140–180
			placebo group (*n* = 11) 4 treatments at 15 days intervals				
Shapiro et al. 2020 [43]	Y	Y	*n* = 35, PRP/placebo on squares 3 PRP treatments (1/month)	36.5 (18–58)	M/F	5	151–170.96
Singh et al. 2020 [44]	Y	Y	PRP group (*n* = 20) 3 treatments (1/month)	26.5	M	3–3.5	93.73–143.2
			PRP+minoxidil group (*n* = 20) 3 treatments (1/month)	25.6	M	3–3.5	90.05–150.45
Tawfick & Osman 2017 [45]	Y	Y	*n* = 30, PRP/placebo on areas 4 PRP treatments (1/week)	29.3 (20–45)	F	0.9	73.66–150.94

The inclusion and exclusion criteria used within the clinical trials selected for analysis are listed in Table S1. Most studies used the Norwood-Hamilton scale for assessment of male patients with AGA and the Ludwig scale for female patients. Exclusion criteria were also largely similar among studies, including the use of other topical or systemic hair growth medications, other causes of alopecia, other general diseases (endocrine, inflammatory, autoimmune, neoplasia, platelet and other bleeding disorders), and risk factors such as alcohol use or smoking.

There were also important differences in the PRP preparation methods, as shown in Table S2. The amount of peripheral blood used for one PRP treatment ranged between 9 and 60 mL, centrifugation was done as a single-step or two-steps, and calcium chloride was the most frequently used agent for α-granule release by platelet fractions. The most widely used monitoring methods were digital photography and phototrichogram analysis using the standard TrichoScan method.

**Figure 2:**
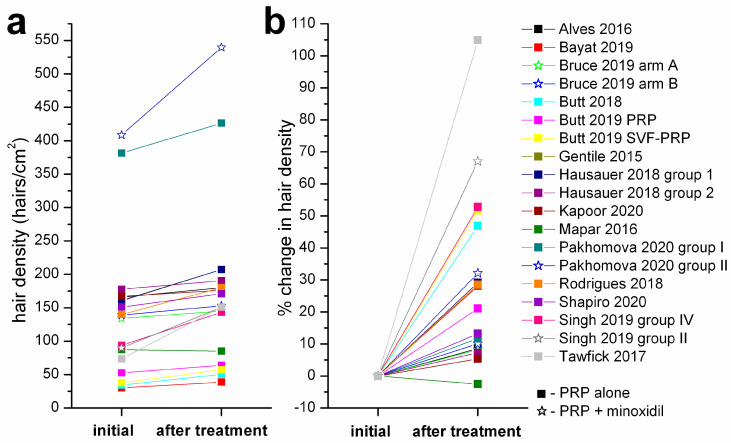
Effects of PRP treatments on absolute and percentage change in hair density within the clinical trials selected for meta-analysis. (**a**) absolute changes in hair density (hairs/cm^2^) at the end of evaluation period. (**b**) percentage changes in hair density relative to initial values. The legend is the same for both graphs. Data for experimental groups where only PRP treatment was applied are marked with squares, those where minoxidil treatment was added are marked with hollow star symbols.

**Figure 3:**
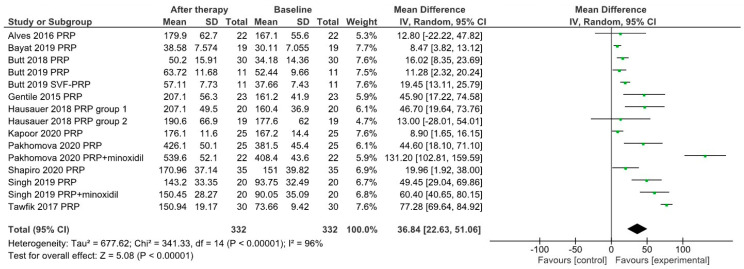
Forrest plot showing the effects of PRP treatment on hair density in selected study groups.

Figure 2 presents the absolute and relative changes in average hair density for *n* = 17 study groups included in this meta-analysis. The mean hair density within these groups varied from an initial value of 141.9 ± 108.2 hairs/cm^2^ (mean ± SD) to 177.5 ± 129.7 hairs/cm^2^ at the last evaluation, a statistically significant increase as proved by a two-tailed Wilcoxon signed rank test (*p* = 0.0004). The same study groups were analyzed for effects on hair density using a Forrest plot shown in Figure 3; the main difference between baseline and post-therapy levels was 36.84 hairs/cm^2^ (95% confidence interval 22.63–51.06).

**Figure 4:**
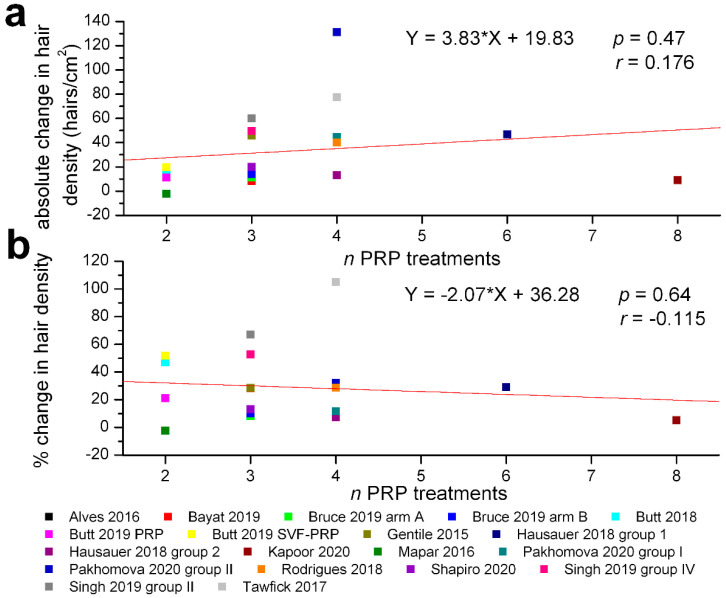
Absence of significant correlations between the number of PRP treatments and absolute or percentage change in hair density within the clinical trials selected for meta-analysis. (**a**) absolute changes in hair density (hairs/cm^2^) at the end of evaluation period. (**b**) percentage changes in hair density relative to initial values (same legend for both graphs). The regression lines are plotted in red, and the corresponding equations, values of correlation coefficients *r* and their probabilities *p* are marked on each graph.

**Figure 5:**
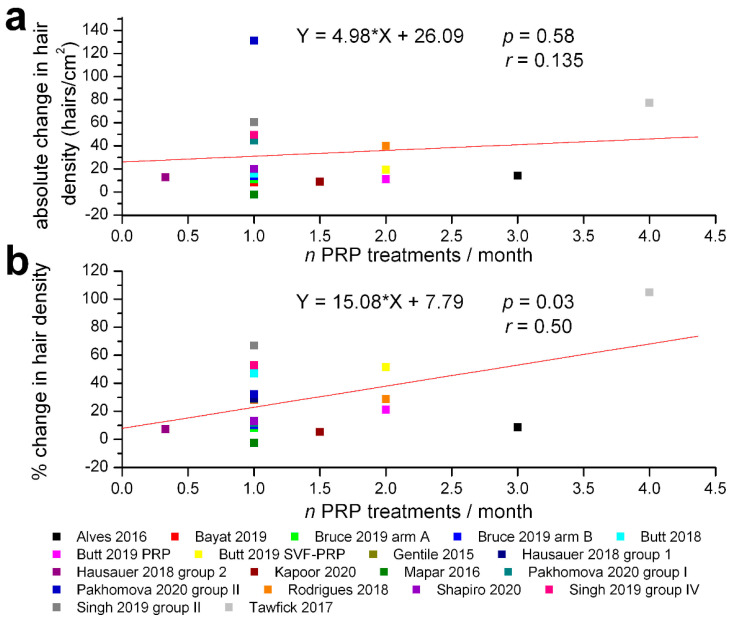
Correlations between the frequency of PRP treatments and absolute or percentage change in hair density within the clinical trials selected for meta-analysis. (**a**) lack of significant correlation of absolute changes in hair density (hairs/cm^2^) at the end of evaluation period. (**b**) significant correlation with percentage changes in hair density relative to initial values (same legend for both graphs). The regression lines are plotted in red, and the corresponding equations, values of correlation coefficients *r* and their probabilities *p* are marked on each graph.

**Figure 6:**
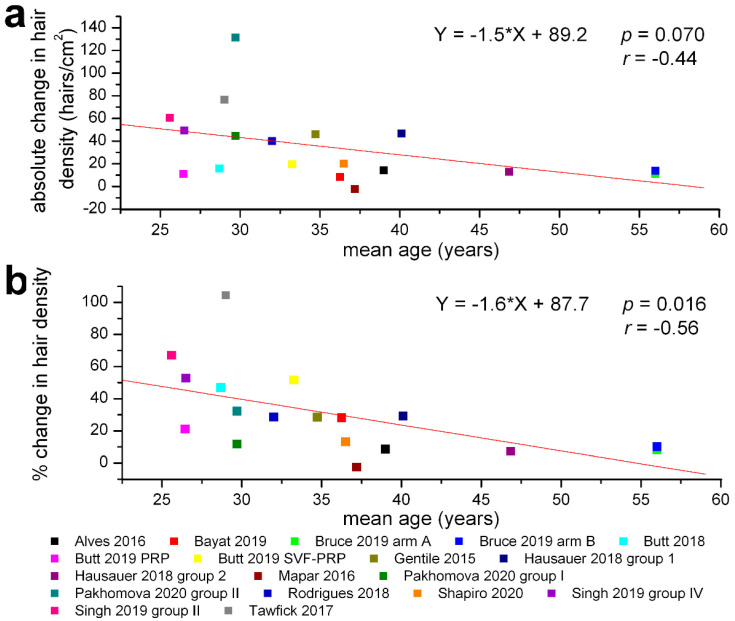
Correlations between the mean age of experimental groups and absolute or percentage change in hair density within the clinical trials selected for meta-analysis. (**a**) almost significant correlation with the absolute changes in hair density (hairs/cm^2^) at the end of evaluation period. (**b**) significant correlation with percentage changes in hair density relative to initial values (same legend for both graphs). The regression lines are plotted in red, and the corresponding equations, the values of the correlation coefficients *r,* and their probabilities *p* are marked on each graph.

We have also assessed the correlations between the total number of PRP treatments and the absolute or relative change in hair density: both were statistically non-significant (r = 0.176, *p* = 0.47 for absolute and r = −0.115, *p* = 0.64 for relative changes, Figure 4). However, the percentage change in hair density was significantly correlated with the frequency of PRP treatments (r = 0.50, *p* = 0.03), but the absolute change in hair density was not (r = 0.135, *p* = 0.58) (Figure 5). We also found no significant correlation between the amount of PRP administered per treatment and the absolute (r = −0.069, *p* = 0.78) or relative change in hair density (r = −0.215, *p* = 0.38), while the mean ages of the treatment groups were significantly correlated with the absolute (r = −0.44, *p* = 0.07) and relative (r = −0.56, *p* = 0.016) change in hair density (Figure 6). 

We verified if the sex composition of study groups exerted an effect on initial values, absolute and relative changes in hair density, using data exposed in Supplementary Table S3. Thus, the initial hair density, the absolute and the relative (%) change in hair density did not show significant differences between study groups formed exclusively from males vs. groups of females or with mixed gender composition.

**Figure 7:**
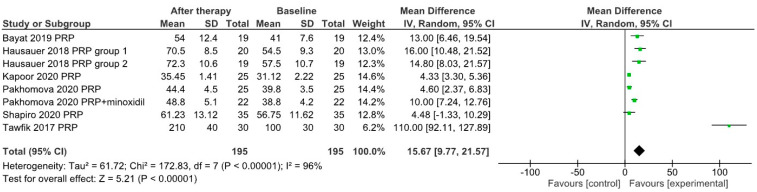
Forrest plot showing the effects of PRP treatment on mean hair diameter in selected study groups.

**Figure 8:**
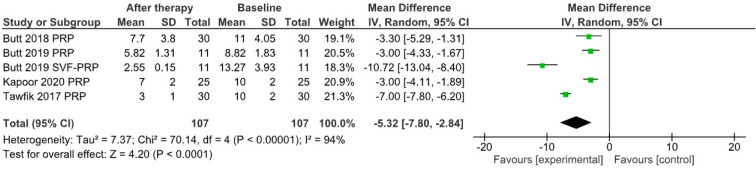
Forrest plot showing effects of PRP treatment on results of pull test in selected study groups.

Although relatively few studies of those included in the meta-analysis provided relevant data, we assessed the effects of PRP therapy on the mean hair diameter and the results of the pull test. Therefore, for n = 8 study groups the mean hair diameter increased by 15.67 µm after therapy (95% confidence interval 9.77–21.57) (Figure 7), while the results of the pull test decreased on average by 5.32 (95% confidence interval 2.84–7.80) for n = 5 study groups (Figure 8).

8 of the 15 studies included in the analysis (53.3%) reported use of patient self-assessment questionnaires, and 5 of them (33.3%) used physician assessments. Patient self-assessment comprised most often the degree of satisfaction following PRP therapy, usually at several time points, and divided on four levels (e.g., highly satisfied, satisfied, dissatisfied, highly dissatisfied). One study reported the percentage improvement after therapy on a 5-level scale [44], and another one on a 1-10 scale [45]. One self-assessment questionnaire included supplementary items such as evaluation of results (Yes, No, Unsure/maybe), recommendation to other patients (Yes, No, Maybe), and motivation to continue (Yes, No, Maybe) [38]. Physician assessments were based on analysis of global photographs of the scalp or four-level satisfaction questionnaires.

## 4. Discussion

AGA is a challenging disorder for both healthcare providers and patients. Current therapeutic options for AGA may generate biochemical abnormalities and clinical adverse effects. Therefore, new personalized therapies are necessary according to the characteristics of the patients, with fewer side effects and satisfactory results [46,47,48,49]. An increasing body of evidence emphasizes an important dermal and follicular response to certain growth factors such as platelet-derived growth factor, transforming growth factor beta, etc. [17,50,51]. In line with this, recent research has focused on evaluating the effectiveness of PRP in AGA.

The present meta-analysis succeeded in retrieving a sufficiently large number of clinical trials including PRP treatments for AGA in male, female, or mixed gender groups to allow pertinent quantitative analysis of results. By comparison, a relatively recent similar meta-analysis, Giordano, Romeo and Lankinen (2017) [3], found only 6 out of 16 clinical trials of PRP in AGA accurately reporting changes in hair density following treatment. A similar attempt of meta-analysis, Kramer & Keaney (2018) [52], focusing on technical details of PRP preparation, concluded that only 21% of the 19 studies selected for analysis provided complete quantitative assessment of initial and final cellular composition of PRP preparations. Data reporting seems to have improved in recent clinical trials [17,53], so we were able to retrieve accurate quantitative estimates of changes in hair density obtained with reliable standardized methods in 14 out of 15 studies (Table 1), as well as sufficient details concerning PRP preparation or delivery protocols.

The amount of PRP administered per treatment varied between 0.9 and 10 mL (Table 1 and Table S2), and it was distributed at multiple subcutaneous injection points, each receiving 100–150 µL. Interestingly, some studies reported significant increases in hair density even for the placebo treatment (e.g., from 151.04 ± 41.99 to 166.72 ± 37.13 hairs/cm^2^, mean ± SD [43]). Although the authors explained such effects as resulting from a possible diffusion of the growth factors from the PRP-injected to the placebo-injected area, it is also plausible that microstimulation by needle puncture itself exerts beneficial effects on hair growth; this principle is currently applied in microneedling, another novel therapy used for AGA [54,55].

Among the 15 clinical trials selected for this meta-analysis, only two included histopathological and/or immunohistochemical assessment of PRP treatment effects. Gentile et al. (2015) showed significant increases in epidermal thickness and number of hair follicles after PRP injection on hematoxylin-eosin-stained scalp biopsies; they also assessed Ki67 proliferation index of hair follicle bulge cells and basal cells of epidermis [37]. Pakhomova and Smirnova (2020) proved by immunohistochemistry post-PRP treatment increases in areas of CD34 and β-catenin reactivity, as well as almost doubling of Ki67 proliferative index [40]. Rodrigues et al. (2018) used the multiplex method (Luminex^®^) to assess VEGF, PDGF and EGF concentrations in PRP preparations and correlate with their platelet counts, but also showed a lack of correlation with clinical effects on hair density [42]. 

Although there is no consensus concerning the molecular mechanisms and signaling pathways involved in PRP therapy, histological examination of biopsy samples from treated vs. control areas and immunohistochemical markers suggest multiple changes such as increased epidermal cell proliferation, particularly at hair follicles dermal papillae, augmentation of blood capillary network, and strengthening of the basal dermal extracellular matrix [43]. Platelet-secreted factors present in PRP preparations seem to enhance hair growth by stimulating multiple signaling pathways involved in the hair follicle cell cycle, such as the protein kinase B (Akt) pathway [18]. Upon Akt phosphorylation, subsequent Wnt/β-catenin transcription upregulation induces stem cell differentiation into hair follicle cells [56]. In vitro studies also showed that PRP causes papillary cells of the skin to grow by activating the extracellular signal-regulated kinases (ERK) [49]. Another important effect of PRP is reduction of hair follicle microinflammation associated with hair loss during AGA [1,57]. PRP growth and transcription factors signal the follicle to enter the anagen versus catagen phase, playing a role in regeneration and renewal; these effects partly overlap those of other effective AGA therapies such as minoxidil [58] or finasteride [59], justifying combined therapy approaches [44].

Due to these remarkable properties and activation of multiple signaling pathways by platelet-secreted factors, PRP raised a lot of interest as a potentially beneficial therapy in various medical fields, such as dermatology and cosmetology, ophthalmology, neurology, sports medicine, stomatology, gynaecology and reproductive medicine. A similar remedy is PRF, which is even easier to prepare in either leukocyte-rich or leukocyte-poor varieties, and includes cytokines and growth factors that are released for up to several weeks, resulting in enhanced wound healing [60,61]. 

Our study presents a number of limitations, such as those related to the extent of literature search, as well as the lack of accurate reporting of quantitative assessment variables and details concerning parameters of study groups, particularly in earlier published clinical trials. There is also a high degree of variability concerning PRP preparation and administration protocols, selection of experimental groups and general design of the trials, objective assessment methods. Possibly standardization requirements and larger patient samples will facilitate comparisons between objective outcomes of future studies.

## 5. Conclusions

PRP represents a relatively new therapeutic approach widely used in different areas of dermatology and plastic surgery [19,21,22], although its underlying mechanisms of action are not completely understood. The present meta-analysis proves the valuable role of PRP therapy in AGA for patients of both sexes, but also points to the need for personalized therapeutic indications and approaches. Although the total number of PRP treatments and the amount of PRP injected per treatment do not appear to influence the outcome, an increased frequency of application (number of treatments per month) results in larger increases in hair density. Other important factors are the age of patients and implicitly duration since alopecia onset. For best results it is advisable to apply a complex combined therapy protocol as early as possible.

## Data Availability

Not applicable.

## Supplementary Materials

The following are available online at https://www.mdpi.com/article/10.3390/jpm12030342/s1, Table S1: Inclusion and exclusion criteria for the clinical trials included in the meta-analysis, Table S2: Methods of PRP preparation, injection and of patient monitoring for the clinical trials included in the meta-analysis, Table S3: Influence of sex composition of study groups on hair density (initial and after PRP treatment).

## Abbreviations

AGA	androgenic alopecia
CASP7	caspase 7
CD	cluster of differentiation
CTGF	connective tissue growth factor
EGF	epidermal growth factor
ERK	extracellular signal-regulated kinases
FDA	United States Food and Drug Administration
FGF	fibroblast growth factor
KGF	keratinocyte growth factor
PDGF	platelet-derived growth factor
PPP	platelet-poor plasma
PRF	platelet-rich fibrin
PRISMA	Preferred Reporting Items for Systematic Reviews and Meta-Analyses
PRP	platelet-rich plasma
ROS	reactive oxygen species
TGF	transforming growth factor
TNF-α	tumor necrosis factor α
VEGF	vascular endothelial growth factor
