# Supplementary material for: Effectiveness of Platelet-Rich Plasma Therapy in Androgenic Alopecia—A Meta-Analysis
Source: J Pers Med. 2022 Feb 24;12(3):342. doi: 10.3390/jpm12030342 (PMC8953144; doi:10.3390/jpm12030342)
Supplement: Supplementary file 1 [file jpm-12-00342-s001.zip › jpm-1602760-supplementary.pdf]

Supplementary materials

Table S1. Inclusion and exclusion criteria for the clinical trials included in the meta-analysis.

Study	Inclusion criteria	Exclusion criteria
Alves & Grimalt 2016	Men aged 18-65 with AGA with Hamilton-Norwood patterns II-V<br>Women aged 18-65 with AGA with Ludwig stage I-III	Use of any topical (minoxidil, etc.) or oral (finasteride, etc.) medication for hair growth during last 12 months<br>Bleeding disorders, platelet dysfunction syndrome<br><150,000 platelets/μL<br>Anticoagulant therapy/NSAIDs during previous 2 weeks<br>Smokers >20 cigarettes/day<br>Pregnancy, lactation<br>Chronic active scalp conditions other than AGA<br>History of hair transplant
Bayat *et al*. 2019	Men with AGA with Norwood-Hamilton grade III-V	topical/systemic medication for AGA<br>retinoids or corticosteroids during last 6 months<br>thyroid or collagen vascular diseases<br>chemotherapy<br>other types of alopecia<br>smoking, alcohol intake, malnutrition
Bruce *et al*. 2019	Females aged 18 or older with AGA with Ludwig stages I-II	Use of any topical (minoxidil, corticosteroids, retinoids etc) or oral (antiandrogens, etc) medication for hair grow during last 3 months<br>Bone marrow aplasia<br>Platelet disorders, antiaggregating, and anticoagulant therapy<br>Sepsis, cancer, imunosupression<br>Uncompensated diabetes mellitus or propensity for keloid formation<br>Pregnancy
Butt *et al*. 2018	Males aged ≥15 years with AGA type III-VI (Hamilton-Norwood scale) and in females type I-III (Ludwig scale)<br>Patients having increased hair loss during the last 12 months	Patients with pregnancy, chronic disease like diabetes, any malignancy, thinning of scalp hair globally or in occipital areas<br>Inflammation or any type of infection on scalp<br>Anticoagulant medication during last 5 years
Butt *et al*. 2019	Male / female patients with androgenetic alopecia aged 15 or older with active hair loss within last 12 months<br>Hamilton score III-VI in male patients<br>Ludwig score I-III in female patients	Patients with inflammation, infection, malignancy, allergic or autoimmune diseases, pregnancy,<br>Current anticoagulant therapy<br>Chemotherapy during last 5 years
Gentile *et al*. 2015	Men aged 19-63 with AGA stage Iia-IV (Norwood-Hamilton classification)	Topical/systemic medication for AGA during the previous 12 months<br>Platelets disorders, thrombocytopenia, antiaggregating therapy<br>Bone marrow aplasia, imunosupression<br>Uncompensated diabetes, sepsis, or cancer<br>Propensity for keloids
Hausauer & Jones 2018	Men / women aged 18-60 with AGA stages Norwood-Hamilton II–V / Ludwig I2–II1<br>Subjects whose hair had been clinically stable on FDA-approved AGA therapies (topical minoxidil and/or oral finasteride for 12 months) were allowed to participate without changing their regimens	Non-AGA hair loss, active skin diseases / infections<br>Cuts or abrasions on the scalp<br>History of surgical hair restoration<br>Current or recent malignancy, excluding non-scalp non-melanoma or melanoma skin cancers<br>Current or recent chemotherapy or radiation<br>History of thyroid dysfunction, autoimmune disorders, or blood-borne infections (i.e. HIV, hepatitis B or C)<br>Tendency to develop keloids<br>Anticoagulant therapy, except for aspirin, NSAIDs,

		or vitamin E if discontinued 7-14 days prior to each session; hematologic or coagulation disorders
Kapoor *et al*. 2020	Male patients, aged 25-50 years with Norwood Hamilton grade II-IV<br>Individuals who have not responded to topical minoxidil for a period of 1 year or more<br>Nonresponders of oral finasteride 1 mg for 1 year	History of hair loss <6 months.<br>Patients with serious drug allergies, diagnosed or suspected malignancies, autoimmune/hematologic disorders. Seborrheic dermatitis.<br>Patients who had recently started or stopped oral finasteride and/or minoxidil
Mapar *et al*. 2016	Men aged 25–45 years with AGA grade IV–VI of Norwood hair loss classification, who had not received any treatment in the past three months.	Infection or seborrheic dermatitis on the scalp<br>Coagulation disorders or administration of aspirin during the past three weeks<br>Systemic diseases, hepatitis, HIV infection
Pakhomova & Smirnova 2020	Men aged 18-53 with presence of AGA degrees I-IV on the Norwood-Hamilton scale inclusively	AGA degree VI or higher (Norwood–Hamilton scale)<br>Hyperandrogenism<br>Inflammatory processes on the scalp<br>Use of hair growth-stimulating drugs, androgen synthesis inhibitors (finasteride, dutasteride)<br>Endocrinopathies that affect the exchange of steroids Patients with hair transplant<br>Drugs lowering platelet levels (aspirin/other NSAID)<br>Leukocytosis / thrombocytopenia<br>Presence of side effects from treatment<br>Tendency to keloid / hypertrophic scars
Puig *et al*. 2016	Females at least 18 years of age, diagnosed with Ludwig II female androgenetic alopecia	Other hair loss treatments during the study and for 60 days before the study<br>Without known disease
Rodrigues *et al*. 2020	Men aged between 18 and 50 years and presentation of AGA-III-vertex profile according to the Norwood-Hamilton scale	Previous hair transplantation and other history of any disease related to hair loss, such as thyroid disease and/ or iron deficiency; and present or past neoplasia, kidney, liver, infectious, hematologic, or rheumatoid diseases.<br>Using of antiplatelet and/or antiinflammatory drugs
Shapiro *et al*. 2020	Men aged 18-58 years with Norwood-Hamilton Stage III-V M-AGA<br>Females aged 18-58 years with Ludwig Stage I or II F-AGA<br>Subjects taking aspirin or other NSAIDS were allowed to participate after discontinuing use seven days before beginning treatment. Subjects taking vitamin E supplements were also allowed to participate provided use was discontinued 14 days before beginning treatment	Topical/systemic medication for AGA in the previous 3 months<br>History of hair transplantation, facial cancer, chemotheraphy<br>Hematologic/coagulation disorders, and hemodynamic instability, anticoagulant therapy<br>Any acute infection or autoimmune disease<br>Pregnant or breastfeeding
Singh *et al*. 2019	Male patients aged 18–60 with male-type baldness grade II–V (Hamilton-Norwood scale)	Using topical/oral medication for hair loss during the previous 6 months<br>Inflammation or erythema (except mild seborrheic dermatitis), or scarring over the scalp<br>Anticoagulants, antihypertensives, anticonvulsants, aspirin / other NSAID<br>History of malignancies, bleeding disorders, bone marrow aplasia, diabetes, HIV, hepatitis B or C, imunosupression, propensity for keloids
Tawfick & Osman 2020	Females aged 20-45 with female pattern hair loss grade I-III (Ludwig classification)	Topical or systemic treatments for hair loss in the previous 3 months<br>Patients who are pregnant or those with a present history of keloids, malignancies, bleeding disorders, and thyroid dysfunction

Table S2. Methods of PRP preparation, injection and of patient monitoring for the clinical trials included in the meta-analysis.

Study	PRP preparation and injection method	Monitoring method
Alves & Grimalt 2016	18 ml peripheral blood on 2 ml 3.8% Na citrate centrifuged at 460g 8 min<br>collection of middle PRP layer (3 ml)<br>activation with 0.15 ml $CaCl_2$ before application<br>injection in 4 selected scalp areas 0.15 ml/cm^2 with a 30-G needle, without local anesthesia	Global photography and phototrichogram<br>Global photographs of 3 scalp areas (vertex, frontal, occipital) taken with Canon Canfield Orthostatic Dev. (Omnia Digital Imaging System, Fairfield, NJ)<br>Phototrichograms via epiluminescence microscopy, Digital image analysis with FotoFinder,TrichoScan professional version
Bayat *et al*. 2019	15 ml blood collected from brachial vein on citrate centrifuged 8 min at 2000g<br>PRP centrifuged 15 min at 4000g to collect precipitated platelet-rich fraction<br>5 ml PRP injected at 125 points (0.04 ml/cm^2) under local anesthesia	Digital photography and dermoscopy photos in fixed locations (at equal distance above eyebrows aligned to midpupillary line)<br>Estimates of number of hair follicles per unit area and average hair thickness<br>Clinical assessment by 2 independent observers using the 15-point Jaeschke scoring system
Bruce *et al*. 2019	60 ml blood collected from peripheral vein using 8 ml citrate dextrose solution as anticoagulant<br>Initial 10-minute centrifugation (1500 rpm) with additional centrifugation (3500 rpm) for 10 minutes<br>5 ml PRP injected at 50 injection points (0.1 ml/point) with 30-G needle under cold air analgesia	Standardized TrichoScan analysis and quality-of-life questionnaires were assessed at baseline and 12-week follow-up for each treatment
Butt *et al*. 2018	9 ml of blood with sodium citrate centrifuged at 1000 rpm for 10 min.<br>PRP injected into affected scalp areas (0.05-0.1 ml/cm^2) at 1 cm distance at a depth of 1.5-2.5 mm	Macroscopic photographs, pull test, trichoscopic photomicrographs, physician global assessment score (PhGAS), and patient global assessment score (PaGAS)
Butt *et al*. 2019	9 ml whole blood collected on sodium citrate centrifuged 10 minutes at 650 g to obtain 5 mL of PRP; intradermal injection with insulin syringe in points at 0.5 cm apart under lignocaine gel anesthesia	Macroscopic photographs with Nikon camera<br>Trichoscan by standard epiluminescence microscopy<br>for hair density; Pull test; Physician global assessment score (PhGAS) by 2 evaluators
Gentile *et al*. 2015	Cascade-Selphyl-Esforax system (18 ml blood) or Platelet Rich Lipotransfert system (60 mL peripheral blood) collected using Na citrate centrifuged at 1,200 rpm for 10 min; platelet secretion stimulated by Ca^{2+}<br>9 mL PRP (0.1 ml/cm^2) injected in selected areas 3 times at 30-days intervals, without anesthesia	Standardized phototrichograms with Fotofinder video-epiluminescence microscopy and Trichoscan digital image analysis<br>Global photography, physician's and patient's global assessment scale<br>3-mm punch biopsies, hematoxylin-eosin stain and immunohistochemistry (Ki67)
Hausauer & Jones 2018	EclipsePRP kit: 22 ml peripheral blood centrifuged at 3500 rpm for 10 min<br>4-6 mL PRP injected in 0.2 to 0.5 mL aliquots sub-dermally using a 32-G half-inch needle every 2 to 3 cm at balding areas; optional topical lidocaine anesthesia	Baseline magnified Folliscope2.8 and global (Hair Metrix) photographs for assessment of hair count (hairs/cm^2), shaft caliber (μm), and Norwood–Hamilton or Ludwig scale<br>Satisfaction and outcome questionnaires
Kapoor *et al*. 2020	1.5 mL of PRP were used in a total of 8 sessions at 3-weeks intervals<br>60-70 tiny (0.02 mL) intradermal injections were administered at points located 1 cm apart, covering the visible areas of hair thinning and alopecia	All patients evaluated at baseline<br>Standard global photographic and videometric assessment<br>Hair pull test<br>Patient self-assessment
Mapar *et al*. 2016	9 ml of blood from antecubital veins on 1 ml acidic citrate dextrose; centrifuged 6 min at 3000 rpm;<br>recentrifuged 3 min at 3300 rpm<br>Dermal injection of 1.5 mL PRP with 30-G needle in 2 sessions (1 month apart) using acidic citrate dextrose and 0.1 ml of calcium gluconate for every ml of PRP	Terminal and vellus hairs counted in each square before every injection using a magnifying glass

Pakhomova & Smirnova 2020	18 ml venous blood collected on 3.8% sodium citrate (10:1 ratio); centrifuged 5 min at 570g; recentrifuged 10 min at 1200g; $CaCl_2$ 1:20 used as activator<br>4 procedures at 1-month intervals: 4 ml of PRP injected intradermally (0.15 ml per injection) in the parietal zone of AGA	Trichological study with a digital video camera “Aramo S” and the TrichoSciencePro v 1.3RUS computer program (control points marked with a tattoo); hair density (cm^2),proportion of vellus and telogen hairs (%), average diameter of hairs (μm)<br>Immunohistochemistry of skin biopsies for Ki67, β-catenin, CD34, DKK-1
Puig *et al*. 2016	Angel PRP system (Cytomedix): 10 ml PRP from 60 ml blood (2.75-3.4x platelet concentration factor) 1 treatment in an area of 10 cm located in the central scalp	Photography (for hair count)<br>Cohen hair check system (for hair mass index)<br>Patient survey
Rodrigues *et al*. 2020	50 ml peripheral vein blood collected on acid citrate dextrose, 4 ml on EDTA and 1 ml without anticoagulant (autologous serum used as activator); centrifuged 15 min at 1258g<br>4 applications at 15-days intervals<br>2 mL PRP/saline applied with 32-G needle in 20 subcutaneous injections of 100 μl into the scalp	TrichoScan method for hair growth, hair density, and percentage of anagen hairs<br>Evaluation before injection, at 15 days and 3 months after last injection<br>Growth factors (PDGF, EGF, and VEGF) measured by Luminex method
Shapiro *et al*. 2020	Regen Blood Cell Therapy Kit: 10 ml blood from antecubital veins; centrifuged 5 min at 1500g in tubes with thixotropic gel<br>3 treatment sessions at 1-month intervals<br>5 mL PRP injected (0.1-0.2 ml per injection) at a depth of 3-4 mm, 35°-45° angle; topical lidocaine anesthesia at request	Folliscope examination (hair density, mean hair shaft diameter)<br>Photography at baseline and each subsequent visit<br>Investigator Global Assessment questionnaire<br>Self-assessment by Quality of Life Questionnaire
Singh *et al*. 2019	18 ml blood collected on 2 ml sodium citrate 3.8% centrifuged 10 min at 2200rpm; recentrifuged 6 min at 3000rpm (4.2x platelet concentration factor); platelet secretion activated with calcium gluconate<br>3 monthly sessions with 3-3.5 ml PRP intradermal (0.05-0.1 ml/cm^2) following topical anesthesia	Dermoscopic photographs of a 1-cm^2 area taken at fixed site (repeated every month) (for hair density)<br>Clinical photography<br>Patient self-assessment (monthly for 5 months)
Tawfick & Osman 2020	10 ml blood from median cubital vein collected on 1.5 ml sodium citrate; centrifuged 15 min at 1200g;<br>recentrifuged 10 min at 2000g; calcium gluconate 1:9 added to PRP; 4 treatments at 1-week intervals	Global photography, standardized phototrichograms,<br>hair pull test, patient’s satisfaction scale

Table S3. Influence of sex composition of study groups on hair density (initial and after PRP treatment)

**Study**	**Sex**	**Hair density in control group (hairs/cm^2)**	**Absolute change in hair density (hairs/cm^2)**	**Relative change in hair density (%)**
Bayat *et al*. 2019	M	30.11	8.47	28.13
Gentile *et al*. 2015	M	161.2	45.9	28.47
Kapoor *et al*. 2020	M	167.2	8.9	5.32
Mapar *et al*. 2016	M	87.29	-2.23	-2.55
Pakhomova & Smirnova 2020 group I	M	381.5	44.6	11.69
Pakhomova & Smirnova 2020 group II	M	408.4	131.2	32.13
Rodrigues *et al*. 2020	M	140	40	28.57
Singh *et al*. 2019 PRP group	M	93.73	49.47	52.78
Singh *et al*. 2019 PRP+minoxidil group	M	90.05	60.4	67.07
**Mean ± SD**		**173.28 ± 132.79**	**42.97 ± 39.60**	**27.96 ± 22.00**
Alves & Grimalt 2016	M/F	165.7	14.2	8.57
Bruce *et al*. 2019 arm A	F	134	11	8.21
Bruce *et al*. 2019 arm B	F	139	14	10.07
Butt *et al*. 2018	M/F	34.18	16.02	46.87
Butt *et al*. 2019 PRP	M/F	52.64	11.08	21.05
Butt *et al*. 2019 SVF-PRP	M/F	37.66	19.45	51.65
Hausauer & Jones 2018 PRP group 1	M/F	160.4	46.7	29.11
Hausauer & Jones 2018 PRP group 2	M/F	177.6	13	7.32
Puig *et al*. 2016	F			
Shapiro *et al*. 2020	M/F	151	19.96	13.22
Tawfick & Osman 2020	F	73.66	77.28	104.91
**Mean ± SD**		**112.58 ± 56.60**	**24.27 ± 21.36**	**30.10 ± 30.86**
**Statistical significance of differences between M and F/mixed groups**		$t = 1.321$<br>17 *d.f.*<br>$p = 0.204$	$U = 38.00$<br>$p = 0.604$	$t = 0.1723$<br>17 *d.f.*<br>$p = 0.865$
